# Altered expression response upon repeated gene repression in single yeast cells

**DOI:** 10.1371/journal.pcbi.1010640

**Published:** 2022-10-18

**Authors:** Lea Schuh, Igor Kukhtevich, Poonam Bheda, Melanie Schulz, Maria Bordukova, Robert Schneider, Carsten Marr

**Affiliations:** 1 Institute of AI for Health, Helmholtz Zentrum München—German Research Center for Environmental Health, Neuherberg, Germany; 2 Institute of Functional Epigenetics, Helmholtz Zentrum München—German Research Center for Environmental Health, Neuherberg, Germany; 3 Institute of Computational Biology, Helmholtz Zentrum München—German Research Center for Environmental Health, Neuherberg, Germany; 4 Department of Mathematics, Technical University of Munich, Garching, Germany; 5 German Center for Diabetes Research (DZD), Neuherberg, Germany; 6 Faculty of Biology, Ludwig-Maximilians University of Munich, Planegg-Martinsried, Germany; University of Connecticut School of Medicine, UNITED STATES

## Abstract

Cells must continuously adjust to changing environments and, thus, have evolved mechanisms allowing them to respond to repeated stimuli. While faster gene induction upon a repeated stimulus is known as reinduction memory, responses to repeated repression have been less studied so far. Here, we studied gene repression across repeated carbon source shifts in over 1,500 single *Saccharomyces cerevisiae* cells. By monitoring the expression of a carbon source-responsive gene, galactokinase 1 (*Gal1*), and fitting a mathematical model to the single-cell data, we observed a faster response upon repeated repressions at the population level. Exploiting our single-cell data and quantitative modeling approach, we discovered that the faster response is mediated by a shortened repression response delay, the estimated time between carbon source shift and Gal1 protein production termination. Interestingly, we can exclude two alternative hypotheses, i) stronger dilution because of e.g., increased proliferation, and ii) a larger fraction of repressing cells upon repeated repressions. Collectively, our study provides a quantitative description of repression kinetics in single cells and allows us to pinpoint potential mechanisms underlying a faster response upon repeated repression. The computational results of our study can serve as the starting point for experimental follow-up studies.

## Author summary

Cells have to continuously adjust to their environment and cope with changing temperature, stress conditions, or metabolic resources. Yeast cells exposed to repeated carbon source shifts have shown to be “primed” by their first exposure, exhibiting enhanced gene expression of specific genes later on. However, how cells respond to a repeated repressive stimulus, e.g., withdrawal of metabolic resources, has been so far much less studied.

For this, we investigated the expression kinetics of a carbon source-responsive gene across repeated repressions. We measured single-cell expression and used mathematical modeling to evaluate potential causes underlying an observed faster repression response upon a repeated stimulus. Specifically, we investigated whether i) an increased dilution due to e.g., proliferation, ii) an increased fraction of repressing cells, or iii) different kinetic parameters in the repeated repression cause the observed faster response in the second repression. Leveraging quantitative mathematical model comparison, we discovered that the faster response is mediated by a shortened estimated time between carbon source shift and protein production termination at the single-cell level.

Our study provides a quantitative description of repression kinetics in single cells and allows us to pinpoint potential mechanisms underlying a faster response upon repeated repression.

## Introduction

Cells receive and process external signals to optimally adjust to changing environments. Repeated stimulation from the same external signal induces an altered transcriptional response, a phenomenon termed transcriptional memory [1], with implications for a broad range of cellular functions, including the human adaptive immune system [2,3], disease development in diabetes [4,5], and aging [6]. So far, transcriptional memory has primarily been studied with respect to gene induction, despite gene repression playing an essential role in gene regulation [7,8].

The adjustment of *Saccharomyces cerevisiae* (budding yeast) to carbon sources is among the most well-studied eukaryotic signal integration systems. Whereas glucose directly enters glycolysis, a vital metabolic route providing cells with energy, galactose is first converted to glucose-6-phosphate [9,10], necessitating the production of Gal gene-encoded enzymes [11]. Repeated alternations between glucose and galactose media revealed that yeast cells are primed by their carbon source history, exhibiting transcriptional memory: repeated galactose induction results in enhanced Gal gene expression [12–16]. In Bheda et al. we examined the expression of the galactokinase 1 gene (*Gal1*) in single cells, for which reinduction memory has been well characterized, and discovered that a shorter delay, rather than an increased expression rate, contributed to the observed increase in Gal1 levels [17].

In contrast, multiple rounds of transcriptional repression have been much less studied. Lee et al. identified a stronger decrease in transcription levels and a faster response upon repeated galactose exposure in bulk experiments [18]. Here, we systematically analyzed short-term transcriptional repression kinetics in single cells. For this, we studied the expression kinetics of a carbon source-responsive gene across repeated repressions. We used mathematical modeling and the single-cell expression information to evaluate potential causes underlying a faster response. We investigated whether i) an increased dilution due to e.g., proliferation, ii) an increased fraction of repressing cells, or iii) different kinetic parameters in the repeated repression cause the observed faster response in the second repression. Specifically, we used computational methods to deconvolute protein kinetics from dilution effects in single cells and developed a mathematical model to quantitatively describe single-cell repression kinetics and to determine the subpopulation of repressing cells. Additionally, we used the estimated single-cell parameters of protein production, degradation and repression response delay to identify changes in kinetic parameters.

## Results

### Faster Gal1 response upon repeated repression

**Fig 1:**
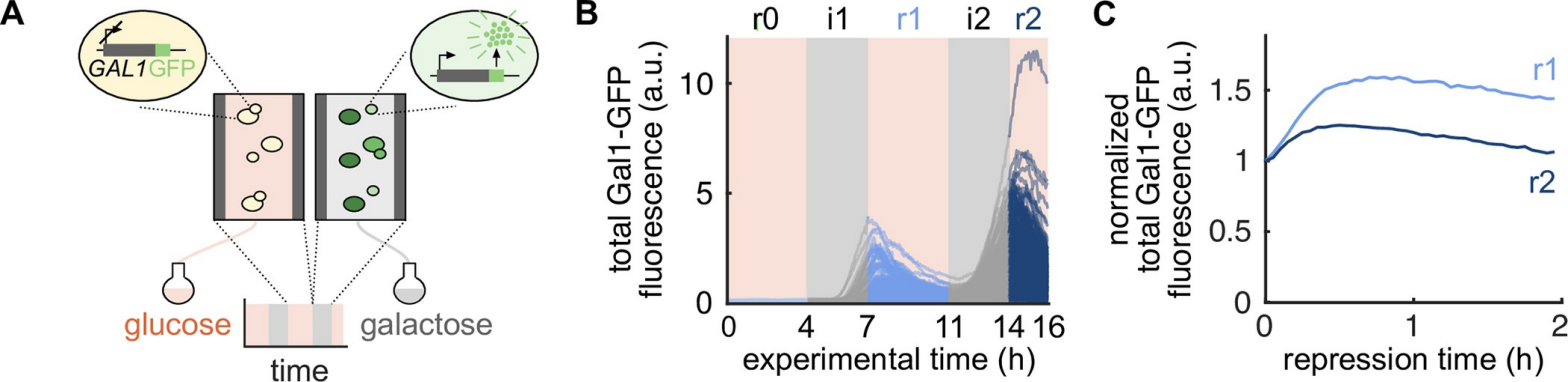
Faster Gal1 response upon repeated repression. (A) Budding yeast cells were grown in microfluidic chambers and alternatingly exposed to a medium containing either glucose (orange) or galactose (gray) as carbon source. Galactokinase 1 (*Gal1*) is induced in cells exposed to galactose and repressed in cells exposed to glucose. Gal1 expression was monitored via a Gal1-GFP fusion and time-lapse microscopy. (B) Single-cell traces of total Gal1-GFP fluorescence signal across two inductions i1 and i2 (gray) and repressions r0, r1, and r2 (blue). (C) Population means of the normalized total Gal1-GFP fluorescence signals of repressions r1 and r2, shifted to the respective start of the repression time.

To study Gal1 protein kinetics over multiple short-term repressions, we re-analyzed images/movies of budding yeast cells from Bheda et al., which were alternatingly exposed to glucose or galactose media (Fig 1A) [17]. For this, cells had been cultured in custom-made microfluidic devices to ensure precise media shifts and long-term tracking. Gal1 expression levels in single yeast cells had been monitored using a Gal1-GFP fusion, a standard reporter to study gene expression in time-lapse microscopy and images from the microfluidics chambers had been taken every 3 min resulting in 320 images per chamber during a 16 h experiment (see Bheda et al., 2020 for details). Semi-automatic segmentation, tracking of the yeast cells and extraction of the total Gal1-GFP fluorescence signal per cell and time point using Autotrack and PhyloCell [19], YeaZ [20] and Cell-ACDC [21] (see Materials and Methods) yielded in over 1,500 single-cell Gal1 expression traces (Fig 1B). To obtain an independent biological replicate, we repeated the experiment and analyzed it according to Bheda et al. (S1 Fig). As expected, the total Gal1-GFP fluorescence signal of single cells recapitulates Gal1 inductions and repressions during galactose and glucose, respectively, and increased overall Gal1 levels in induction i2 in both the original data as well as in our new experiments (Figs 1B and S1A). We also visually observed a faster response of Gal1-GFP fluorescence at the population level upon repression r2 (Fig 1C), which is in line with the findings of Lee et al. [18].

### Dilution effects cannot explain faster response upon repeated repression

**Fig 2:**
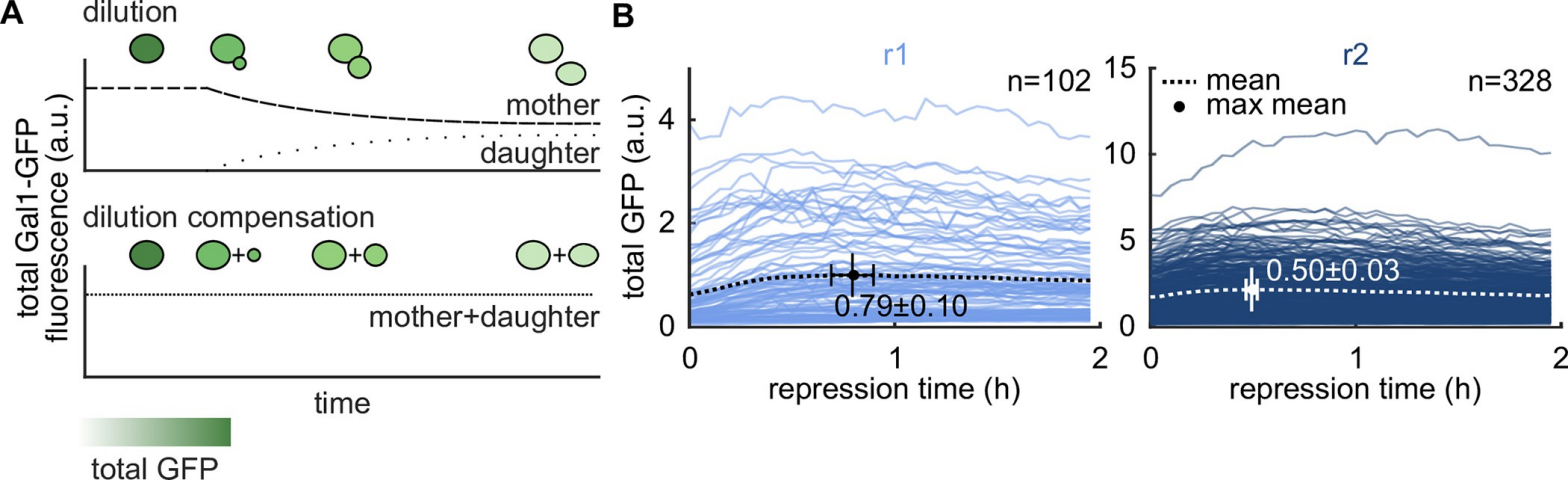
Dilution effects cannot explain the faster response upon repeated repression. (A) Budding decreases the total Gal1-GFP fluorescence signal in mother cells and increases the total Gal1-GFP fluorescence signal in daughter cells (top). To compensate for this dilution, we summed up the total Gal1-GFP fluorescence signal of each mother cell present and its progeny during one repression (bottom). (B) Single-cell traces of total GFP signal adjusted for dilution (see (A)) for the first two hours of repressions r1 (left) and r2 (right). Time to maximal mean total GFP is 17 min shorter in repression r2, where mean total GFP is indicated by the dotted line, the maximal mean total GFP is highlighted by the dot. Bootstrap (10^5^) samples were drawn to generate mean ± std.

During budding, cytoplasmic proteins are disseminated between mother and daughter cells. Assuming a constant Gal1 protein amount, this decreases the total Gal1-GFP fluorescence signal in the mother cell (Fig 2A top), a phenomenon called dilution. Could an increased dilution because of e.g., increased proliferation in repression r2 compared to r1, explain the faster repression response observed upon repeated carbon source shifts (Fig 1C)? The single-cell resolution of our data and asymmetric budding allowed us to identify mother-daughter relationships and to deconvolute dilution and repression kinetics. For this, we calculated the sum of the total Gal1-GFP fluorescence signal of the mother cell, i.e., cell present at the start of repression, and its progeny, i.e., daughter cell(s) detected during repression (Fig 2A bottom, and Materials and Methods) to account for dilution effects due to cell divisions. In the following, the adjusted sum of the total Gal1-GFP fluorescence signal of the mother cell and its progeny is referred to as total GFP. We applied the same dilution compensation to repressions r1 and r2 (Fig 2B). Note that the increase in total GFP traces from n = 102 in r1 to n = 328 in r2 is due to cellular proliferation during the 4 hours of repression r1.

Following a galactose-glucose shift, total GFP intensities initially rise before decreasing. To obtain quantitative insights, we calculated the time to maximal mean total GFP for repressions r1 and r2. This time reduced from 0.79 ± 0.10 h (mean ± std, n = 102 cells) in r1 to 0.50 ± 0.03 h (n = 328 cells) in r2, where the time point of the maximal mean was calculated with10^5^ bootstraps (Fig 2B). A shorter time to maximal mean total GFP suggests a faster response upon repeated repression, even after accounting for dilution effects due to proliferation. Hence, dilution effects cannot explain the faster response in repression r2 at the population level.

### A computational model identifies cells with and without repression kinetics

At the single-cell level, *Gal1* induction delay varies significantly. Zacharioudakis et al. [16] showed that *Gal1* induction caused by a glucose–galactose media shift results in a bimodal population distribution, with only a subset of cells inducing *Gal1* even after several hours of galactose exposure. As in our experiments repression was preceded by 3 h of galactose induction, we expected that our cell population at the start of repression contained induced and uninduced cells, which thus show and do not show protein repression kinetics, respectively. Could a larger fraction of repressing cells in repression r2 explain the faster response observed upon repeated repression (Fig 2B)? To determine the subpopulation of cells with protein repression kinetics, information on single-cell kinetics is required. As it is difficult to distinguish between cells showing and not showing protein repression kinetics from the total GFP traces alone (Fig 2B), we used computational modeling and model selection to systematically describe and classify the kinetics of single total GFP traces. In comparison to other classification methods, model selection allows us to classify total GFP traces without determining arbitrary thresholds for e.g. initial total GFP when classifying induced and uninduced cells at repression start. Since *Gal1* induction results in an approximate 1000-fold change in Gal1 expression [22], we assumed that stochasticity inherent to gene expression was insignificant and that a deterministic modeling approach was sufficient in describing the kinetics of the total GFP traces. To discriminate between cells showing and not showing protein repression kinetics, we defined two models.

**Fig 3:**
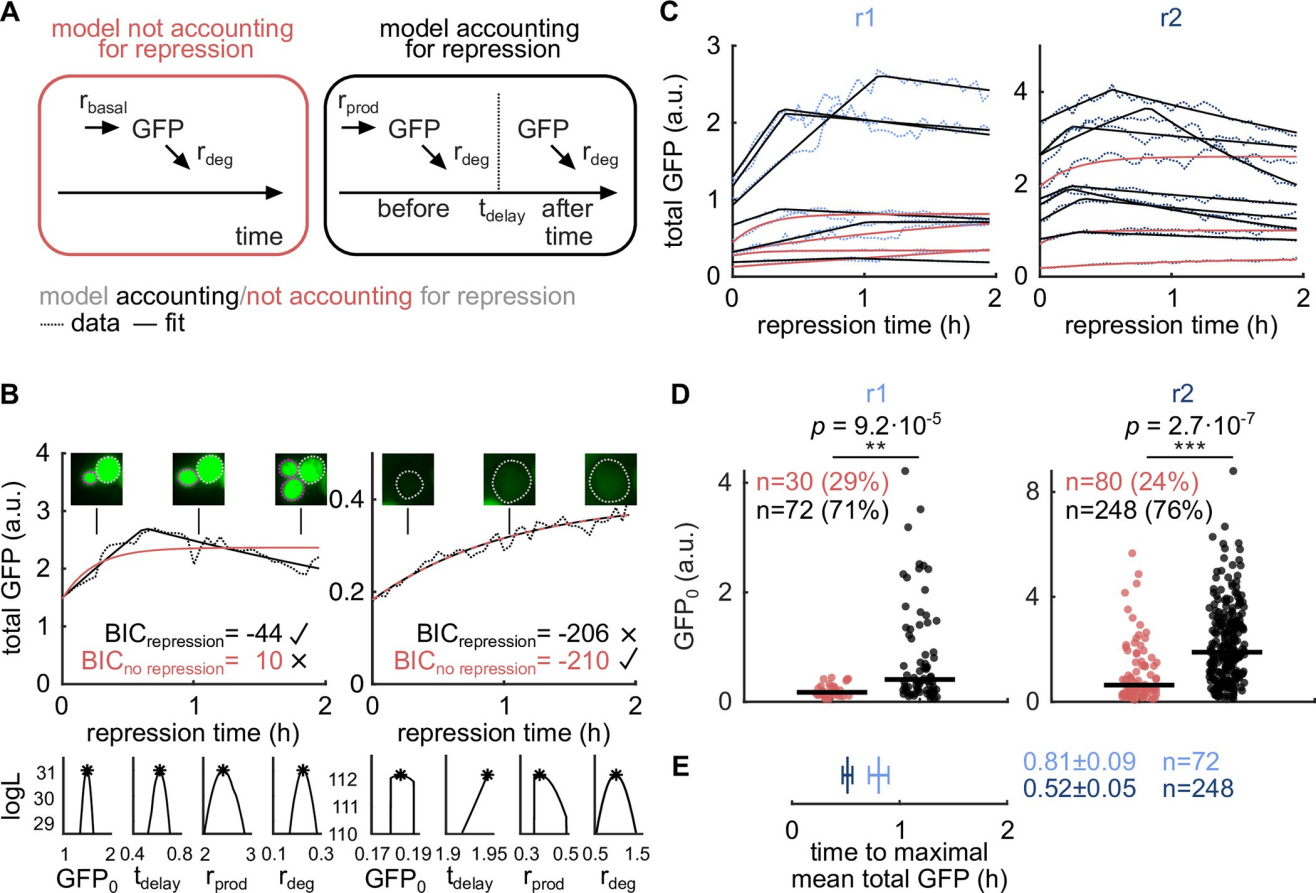
Fraction of cells with repression kinetics cannot explain the faster response upon repeated repression. (A) Left: a model for cells without repression kinetics composed of basal GFP production (r_basal_) and degradation (r_deg_). Right: a model for cells with repression kinetics composed of an initial constant and active GFP production (r_prod_) and degradation (r_deg_) until a delayed repression onset (t_delay_) where GFP production is switched off. (B) Top: two exemplary total GFP traces (dotted line) and fits of the model not accounting for repression (red solid line) and model accounting for repression (black solid line). Exemplary images of the cell(s) at three different time points are shown above. Mother cells are circled in gray, progeny in pink. The better fitting model was selected according to the Bayesian information criterion (BIC). Left: total GFP trace better fitted by the model accounting for repression. Right: total GFP trace fitted equally well by both models. Due to the higher model complexity of the model accounting for repression, the model accounting for repression is rejected. Bottom: profile likelihoods of the model accounting for repression corresponding to the two exemplary total GFP traces above endorse parameter identifiability. Asterisks represent optimized parameters and corresponding log-likelihood (logL) values. (C) Ten exemplary total GFP traces (dotted lines) and best fits (solid lines) for repressions r1 (left) and r2 (right). GFP traces best fitted with the model accounting for repression are shown in black and fits of total GFP traces best fitted with the model not accounting for repression are shown in red. (D) The median initial total GFP, GFP_0_, is significantly higher (*p* = 9.2·10^−5^ and *p* = 2.7·10^−7^, two-sided median test corrected for multiple testing with Bonferroni correction, m = 12) in traces better fitted by the model accounting for repression (black) than in traces better fitted by the model not accounting for repression (red). This confirms that the model accounting for repression fits induced cells better, while the model not accounting for repression fits uninduced cells. The number of cells and percentages of all GFP traces best fitted by the model accounting and not accounting for repression are shown. (E) Time to maximal mean total GFP is decreased in repression r2 for cells with repression kinetics (0.81 h ± 0.09 h vs. 0.52 h ± 0.05 h). Bootstrap (10^5^) samples of the cells with repression kinetics were drawn to generate mean ± std.

The model not accounting for repression assumes a constant basal GFP production and degradation over time with rates r_basal_ and r_deg_ (Fig 3A left), since we observed a gradual increase in total GFP signal in cells visually identified as not showing repression kinetics (Fig 3B top right). Note that r_basal_ encompasses all reaction steps that lead to the production of a detectable GFP protein, including mRNA transcription, Gal1-GFP translation, and GFP maturation. Similarly, r_deg_ comprises active GFP degradation and potential effects of photo-bleaching. Dilution effects are compensated prior to modeling and are not part of r_deg_ (see Methods). The temporal variation of the total GFP signal described by the model not accounting for repression is summarized using the following ordinary differential equation:

$$\frac{\partial \mathrm{GFP}(t)}{\partial t}=r_{\mathrm{basal}}-r_{\deg}\mathrm{GFP}(t).$$

This is solved by

$$\mathrm{GFP}(t)=\frac{r_{\mathrm{basal}}}{r_{\deg}}(1-e^{-r_{\deg}t})+{\mathrm{GFP}}_{0}e^{-r_{\deg}t},$$

where GFP_0_ = GFP(0), the initial total GFP at time point 0.

According to the model accounting for repression, cells that induced Gal1 during galactose induction require time to reestablish glucose-mediated repression. Hence, GFP is actively produced at rate r_prod_, where r_prod_ comprises a basal production rate r_basal_ and an additional production rate accounting for the active production of GFP, till a time point t_delay_. GFP production is switched off (r_prod_ = 0) and GFP is degraded with rate r_deg_ after this estimated repression response delay t_delay_ (Fig 3A right). Again, r_prod_ includes mRNA transcription, Gal1-GFP translation, and GFP maturation, while r_deg_ comprises active GFP degradation, and photo-bleaching, but no dilution. The temporal change of total GFP over time described by the model accounting for repression is summarized by the following ordinary differential equations:

$$\mathrm{before}\;t_{\mathrm{delay}}:\;\frac{\partial \mathrm{GFP}(t)}{\partial t}=r_{\mathrm{prod}}-r_{\deg}\mathrm{GFP}(t)$$

$$\mathrm{after}\;t_{\mathrm{delay}}:\;\frac{\partial \mathrm{GFP}(t-t_{\mathrm{delay}})}{\partial t}=-r_{\deg}\mathrm{GFP}(t-t_{\mathrm{delay}})$$

with solutions

$$\mathrm{before}\;t_{\mathrm{delay}}:\;\mathrm{GFP}(t)=\frac{r_{\mathrm{prod}}}{r_{\deg}}(1-e^{-r_{\deg}t})+{\mathrm{GFP}}_{0}e^{-r_{\deg}t}$$

$$\mathrm{after}\;t_{\mathrm{delay}}:\;\mathrm{GFP}(t-t_{\mathrm{delay}})=\mathrm{GFP}(t_{\mathrm{delay}})e^{-r_{\deg}(t-t_{\mathrm{delay}})},$$

where

$$\mathrm{GFP}(t_{\mathrm{delay}})=\frac{r_{\mathrm{prod}}}{r_{\deg}}(1-e^{-r_{\deg}t_{\mathrm{delay}}})+{\mathrm{GFP}}_{0}e^{-r_{\deg}t_{\mathrm{delay}}}.$$

An example of a cell visually identified as showing repression kinetics is shown in Fig 3B top left. Right next to it, we show the fluorescent microscopy images and total GFP trajectory of a cell visually identified as not showing repression kinetics. Until t_delay_, where t_delay_ < 2 h, the model accounting for repression equals the model not accounting for repression. However, these model definitions allow for model selection and circumvent the usage of arbitrary thresholds for, e.g., the estimated value of t_delay_. Both models comprise four and five model parameters, respectively: initial total GFP, GFP_0_, basal GFP production rate, r_basal_, or GFP production rate, r_prod_, GFP degradation rate r_deg_, a noise parameter σ determining the width of the Gaussian noise distribution (see Materials and Methods), and the repression response delay t_delay_ for the model accounting for repression. For repressions r1 and r2, respectively, we performed multi-start maximum likelihood optimization to estimate the single-cell parameters, and model selection on both models for each total GFP trace (Fig 3B top and 3C). Calculating the profile likelihoods of exemplary total GFP traces, we found the model parameters of the model accounting for repression to be identifiable (Fig 3B bottom). We then determined whether the model accounting for repression was required to explain a total GFP trace using the Bayesian information criterion (BIC). A BIC difference of ten between the model accounting for and not accounting for repression (BIC_repression_ < BIC_no repression_− 10) was considered an appropriate threshold to reject the model not accounting for repression with fewer model parameters (see Materials and Methods and Fig 3B and 3C), as commonly done in model selection using BIC as selection criterion [23–25]. GFP traces of cells requiring the model accounting for repression are henceforth referred to as “cells with repression kinetics”. The median initial total GFP, GFP_0_, was significantly higher in cells with repression kinetics than in cells without repression kinetics (*p* = 9.2·10^−5^ for r1 and *p* = 2.7·10^−7^ for r2), meaning that the model accounting for repression mainly describes cells with higher Gal1 induction levels (Fig 3D). This implies that our models can discriminate between cells that were repressing Gal1 and cells uninduced at the beginning of repression. The overlapping ranges of initial total GFP between cells with and without repression kinetics (Fig 3D) reveal how simple thresholding could result in wrong classification of cells with and without repression kinetics.

### Fraction of cells with repression kinetics in r2 cannot explain faster response upon repeated repression

Using our modeling approach to classify total GFP traces into cells with and without repression kinetics, we found that of all total GFP traces, 71% and 76% of r1 and r2, respectively, require the model accounting for repression (Fig 3D). Since the fraction of cells with repression kinetics is only slightly increased in repression r2, this effect cannot explain the faster response upon repeated repression. Hence, we determined the time to maximal mean total GFP again, this time constraining the analysis to the subpopulation of cells with repression kinetics. We again found a shortened time to maximal mean total GFP in r2, with 0.81 h ± 0.09 h (mean ± std, n = 72 cells) and 0.52 h ± 0.05 h (n = 248 cells) for r1 and r2, respectively (Fig 3E), demonstrating that the faster response in r2 at the population level is not due to the increased fraction of cells with repression kinetics.

### Shortened repression response delay mediates faster response upon repeated repression at the single-cell level

**Fig 4:**
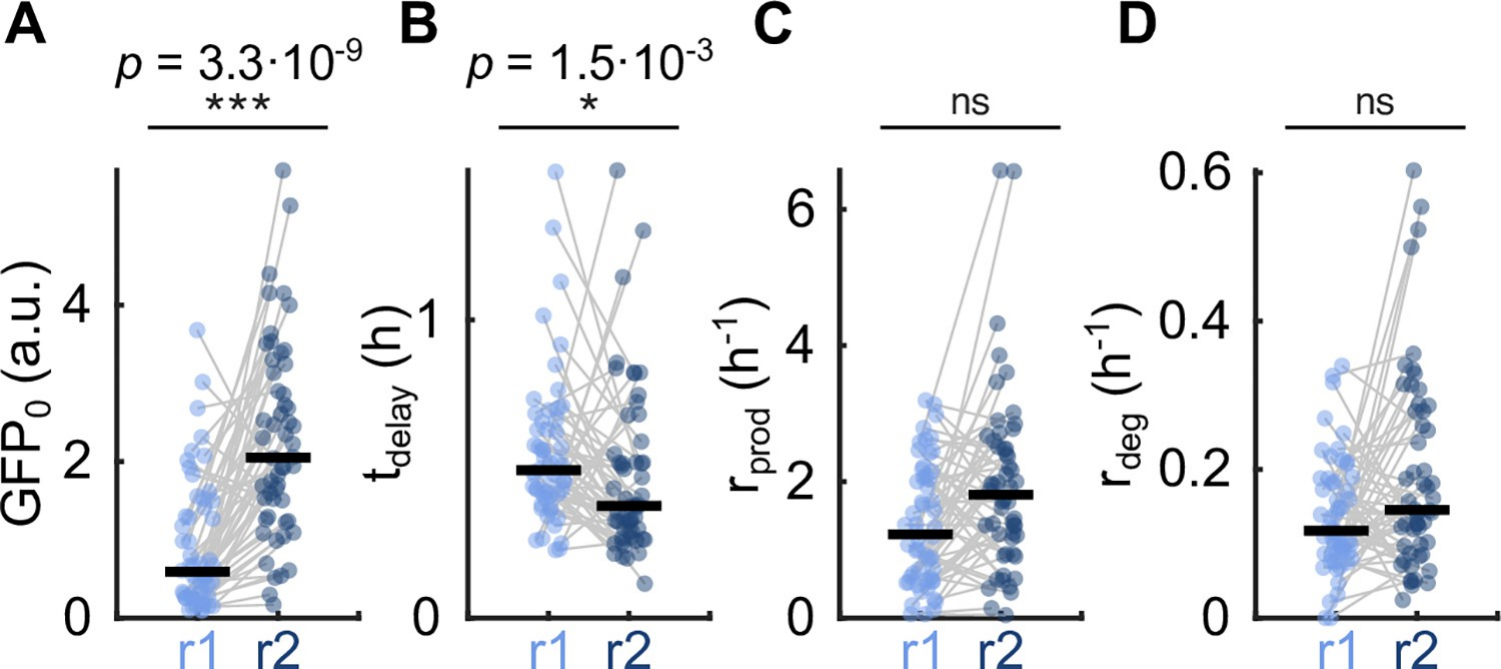
Shortened repression response delay mediates faster response upon repeated repression at single-cell level. Comparison of paired estimated single-cell parameters of cells with repression kinetics of repression r1 and r2 shows that the median initial total GFP, GFP_0_, and median repression delay, t_delay_, are significantly different (*p* = 3.3·10^−9^ and *p* = 1.5·10^−3^, respectively, two-sided paired sign test correcting for multiple testing with Bonferroni correction, m = 12, and the number of paired cells = 54), with median GFP_0_ increased and median t_delay_ decreased (median t_delay_ values of 0.50 and 0.38 for r1 and r2, respectively). GFP degradation is not different between repressions r1 and r2 (*p* = 0.34).

So far, we excluded major contributions of dilution effects and an increased fraction of cells with repression kinetics in r2 to underlie the faster response upon repeated repression. Could altered kinetics of individual cells between repressions r1 and r2 lead to the observed change in repression response? In the previous section, we estimated the kinetic parameters GFP_0_, t_delay_, r_prod_, and r_deg_ for cells with repression kinetics. To compare protein kinetics between repression r1 and r2 at the single-cell level, we used these estimated single-cell parameters of cells with repression kinetics present in both repressions r1 and r2. We discovered that the median initial total GFP, GFP_0_, and median repression response delay, t_delay_, are significantly different (*p* = 3.3·10^−9^ and *p* = 1.5·10^−3^, respectively) between both repressions using a two-sided paired sign test with multiple testing correction (Fig 4A and 4B). Median GFP_0_ is increased (median values of 0.59 and 2.05 for r1 and r2, respectively, Fig 4A), while median t_delay_ is shortened in r2 (median values of 0.50 and 0.38 for r1 and r2, respectively, where 72% of paired cells showed a decrease in t_delay_, Fig 4B). The increased GFP_0_ in r2 conforms to the transcriptional reinduction memory of Gal1 and results in higher GFP_0_ at the start of r2. The median r_prod_ and median r_deg_ between the two repressions were comparable (*p* = 0.22 and *p* = 0.34, respectively) (Fig 4C and 4D). In line with our findings, Bheda et al. reported similar production rates [17]. We repeated the entire analysis based on data from independent experiments (see Methods for details). While the exact parameters vary between replicates due to the heterogeneity in Gal1 induction at the single-cell level, our replicate analysis confirms the conclusions, in particular that a shortened repression response delay in r2 mediates the observed faster response upon repeated repression (S1 Fig). We also tested a more complex, moment equation derived noise model, explicitly accounting for the stochastic nature of single-cell GFP production and degradation (see Methods for details). Due to the increased flexibility of this noise model, we detected fewer cells with repression kinetics for repressions r1 and r2. The set of cells with repression kinetics detected using this revised noise model is, however, largely overlapping with the previously reported set of cells with repression kinetics for both repressions r1 and r2 (S2A and S2B Fig). More importantly, the repression response delays of the cells with repression kinetics detected by both models are estimated to comparable values, suggesting that a more complex noise model does not influence the main conclusions of this work (see S2C and S2D Fig). Of note, a comparison between the estimated single-cell parameters of cells with repression kinetics of the two different models is uninformative, due to the low statistical power of the small set of paired cells with repression kinetics across both models for repressions r1 and r2. Finally, using a simulation study, we tested whether the GFP degradation rate influences our results (S3 Fig). We found that too small degradation rates might lead to misclassifying cells as cells without repression kinetics, hence potentially leading to a loss of information. However, this does not affect our conclusions, since degradation rates are estimated correctly and reliably (S3 Fig).

## Discussion

Here, we described a faster Gal1 response upon repeated short-term repression at the population level in support of what had been shown by Lee et al. [18]. By using single-cell Gal1 expression data and mathematical modeling, we demonstrated that a faster response upon repeated repression is not simply due to increased dilution effects or a larger fraction of repressing cells, but is mediated by a shortened repression response delay at the single-cell level which could suggest the existence of transcriptional repression memory.

We would like to note that we assessed the kinetics of the Gal1-GFP fusion protein, and hence also modeled Gal1-GFP kinetics. Although this reporter has been successfully employed in several studies involving Gal1 gene expression [16,26–29], Gal1-GFP does not allow us to exclude alternative molecular mechanisms driving the shortened repression response delay t_delay_ in repression r2 such as an accelerated mRNA degradation. Altered GFP maturation was accounted for in the production rate r_prod_. Of note, GFP stability is estimated by the degradation rate, r_deg_, which we found to be not significantly different between repressions r1 and r2 (*p* = 0.34). While the degradation rates are estimated to be quite low, they are high enough such that repression kinetics are observable (see Fig 3C). The stability of GFP may only lead to a loss of information, i.e., repression kinetics are overshadowed by the accumulation of GFP, such that cells with repression kinetics might no longer be detected as such (S3 Fig). GFP stability does not influence the estimated repression response delays and hence our conclusions. Potential effects due to photo-bleaching would increase r_deg_ but not affect the estimation of t_delay_ and hence our conclusions.

Due to the rather simple kinetics of the total GFP traces, our model is sufficiently complex to describe the data (Fig 3C). More detailed descriptions of this process e.g., shut off kinetics of protein production, mRNA/promoter kinetics, or histone occupancy, would be desirable but inevitably lead to unidentifiable model parameters and, hence, to little or none additional insight. Similarly, we did not consider cell-size and cell-cycle aware stochastic models, as for example successfully applied by Song et al. [30].

During model design, we also tested mixed effect modeling frameworks, where single-cell parameters are assumed to underlie a certain population distribution and parameter correlations can be accounted for. However, due to the large and unconventional noise distributions underlying the model parameters of our data, mixed effect modeling did not describe the data well enough at the population level. Hence, we believe that for the given data, our simple modeling framework was the most informative approach possible.

Throughout this study, we mainly assumed an additive Gaussian noise model with constant variance to correctly reflect measurement noise. However, other noise models might also be applicable. For example, the Laplacian noise model has shown to be more robust against outliers and, hence, more suitable for corrupted data [31]. We also investigated time-dependent noise from a moment equation based model, taking into account not only measurement but also intrinsic noise emerging from the stochasticity of the underlying molecular processes. While we show that these different noise models lead to the same conclusions in our study, we want to highlight here that identifying a suitable noise model is generally important for both parameter inference as well as model selection [32,33]. The similarity between the two approaches is likely due to the large molecule numbers of Gal1-GFP expression in our study.

In this study, we focused on the altered *Gal1* expression response upon repeated gene repression. Our simple and linear repressor model used to identify the repression delay is basic data analysis, such that its results could be understood as empirical evidence for altered gene repression kinetics. Whether this phenomenon is a unique characteristic of *Gal1* or if this is universal to other non-galactose induced memory genes of, e.g, the MAL or INO system, will be interesting to investigate in the future. Moreover, *Gal1* reinduction memory is inherited by daughter cells [17]. Whether altered repression kinetics can also be maintained through multiple cell divisions is still unknown. Due to yeast cells mainly arresting their cell-cycles in the initial hours of galactose exposure (inductions i1 and i2), dividing cells are rarely detected in i2 and thus we cannot investigate the effect of cell divisions on altered repression kinetics in r2.

Together, our work lays the foundation for further mechanistic studies using single-cell mRNA kinetics or time-resolved information regarding the chromatin architecture at the *Gal1* promoter to investigate the molecular workings underlying a shortened repression response delay upon repeated short-term repression.

## Materials and methods

### Data acquisition and sources

For the analysis for Figs 1 and 2, we used microscopy images and initial segmentation, mapping, and tracking information from a microfluidics experiment from Bheda et al. [17], which contained 13 positions. The images from the first two hours of repression r1 were rectified, and the segmentation, mapping, and tracking were extended to the entire two hours of repression r2. Bheda et al. only segmented r2 partially since they were primarily interested in galactose induction, and did not adjust the final repression frames. Using the software PhyloCell [19], we manually corrected the segmentation, mapping, and tracking of r1 and r2. For the replicate analysis (S1 Fig), we repeated the induction-repression experiment as described in Bheda et al. [17] (Fig 1A). Due to low cell numbers, we pooled data from three independent experiments, totalling 13 positions, respectively. Using the softwares YeaZ [20] and Cell-ACDC [21] for cell segmentation, mapping and tracking, we extracted the relevant single-cell information of the live-cell images for both repressions r1 and r2. During glucose repression, the yeast cells proliferated, increasing the cell numbers within the microfluidic chambers. However, filled microfluidic chambers no longer assure that all the progeny of a cell is recorded, and mapping and tracking of cells become infeasible. To ensure mapping and tracking of single yeast cells within the microfluidic chambers, the glucose repressions were limited to a maximum of 4 h and the overall experiment was limited to 16 h (4 h in glucose (r0), 3 h in galactose (i1), 4 h in glucose (r1), 3 h in galactose (i2), 2 h in glucose (r2)).

### Data preprocessing

We extracted the single-cell information relevant for our analysis, namely cell ID, mother cell ID, detection frame (first frame in which a cell is detected), last frame (last frame a cell is detected), relative GFP intensities per time (mean GFP intensity of a segmented cell) and cell area per time. As the data regarding the relative GFP intensities and cell area was not sorted over time, we first sorted it and then calculated the total GFP fluorescence per time given by

            total GFP fluorescence = relative GFP fluorescence × cell area.

Finally, cells that were not imaged till the end of the experiment, cells with missing relative GFP and/or cell area values, and cells that were supposedly detected before their mother cells (segmentation error) were discarded.

### Dilution compensation

Cytoplasmic proteins are disseminated between the mother and daughter cells during budding. Assuming that Gal1 is not produced or degraded, protein redistribution causes a drop in total Gal1-GFP fluorescence in the mother cell and a rise in total Gal1-GFP fluorescence in the daughter cell till the mother and daughter cells split (Fig 2A top). As a result, regardless of repression, dilution causes variations in total Gal1-GFP fluorescence. The daughter cell grows to about ⅓ of the size of the mother cell [34] such that the decrease in total Gal1-GFP fluorescence due to dilution was expected to be ⅓ of the initial total Gal1-GFP fluorescence of the mother cell. To ensure that dilution does not overshadow potentially more subtle repression kinetics, we created artificial non-dividing cells compensating for dilution by adding the total Gal1-GFP fluorescence of the progeny of a cell present at the start of glucose repression, which we called mother cell, to the total Gal1-GFP fluorescence of that mother cell during the first 2 h of repression (Figs 2A bottom, 2B and S1C). For mother cells with a bud at the beginning of a repression period, we additionally added the bud to the total Gal1-GFP fluorescence of that mother cell. The GFP traces of all computed non-dividing cells can be found under https://github.com/marrlab/Gal1repression. As we found the maximal mean total GFP to be attained before 2 h of glucose exposure, we restricted our analysis to the first 2 h of repression.

### Models

During the first two hours of glucose repression, we modeled the kinetics of the total GFP of every single cell. Due to the high variability in galactose induction, we assumed that our initial cell population at the beginning of repression contained induced and uninduced cells, which show and do not show repression kinetics, respectively. We developed two models, with and without repression, to account for both total GFP kinetics during repression.

### Model not accounting for repression

For more information regarding the model not accounting for repression, see the main text.

### Model accounting for repression

For more information regarding the model accounting for repression, see the main text.

### Noise models

Time-independent noise model taking into account only measurement noise:

Experimental data, such as total GFP per cell per time, is noise corrupted. As a result, we used an underlying additive Gaussian noise model with a constant variance σ^2^ throughout time to test our models. The single-cell specific model parameters are comprised in the parameter vector Θ_i_ for cell i and the experimental measurement at time point k for cell i is denoted by ${\bar{y}}_{i}{}^{k}$. The log-likelihood for the Gaussian noise model is given by

$$\mathrm{logL}(\theta_{i})=\frac{-1}{2}\underset{k}{\sum }\log(2\pi \sigma_{i}^{2})+\frac{{\left({\bar{y}}_{i}^{k}-y(t_{k},\theta_{i})\right)}^{2}}{\sigma_{i}^{2}}.$$

Time-dependent noise model taking into account measurement and intrinsic noise:

Due to the stochastic nature of GFP production and degradation, the single-cell GFP measurements also underlie, in addition to measurement noise, intrinsic noise. The variance of the more sophisticated noise model is hence composed of (1) the constant measurement noise σ^2^ and (2) the time-dependent variance of the stochastic process underlying the corresponding model. According to the moment equations, the time evolution of the variance of the stochastic process underlying the model not accounting for repression is given by

$$\frac{\partial \mathrm{Var}(t)}{\partial t}=-2r_{\deg}\mathrm{Var}(t)+r_{\mathrm{basal}}(2-e^{-r_{\deg}t})+{\mathrm{GFP}}_{0}r_{\deg}e^{-r_{\deg}t},$$

with solution

$$\mathrm{Var}(t)=\frac{1}{r_{\deg}}e^{-2r_{\deg}t}(r_{\deg}({\mathrm{GFP}}_{0}(e^{r_{\deg}t}-1)+{\mathrm{Var}}_{0})+r_{\mathrm{basal}}e^{r_{\deg}t}(e^{r_{\deg}t}-1)),$$

where GFP_0_ = GFP(0), the initial total GFP at time point 0, Var_0_ = Var(0), the initial stochastic variance at time point 0, and GFP(t) as derived in the main text.

For the model accounting for repression, we need to consider the piecewise stochastic processes before and after t_delay_. The time evolutions of the variances of the stochastic processes are given by

$$\mathrm{before}\;t_{\mathrm{delay}}:\;\frac{\partial \mathrm{Var}(t)}{\partial t}=-2r_{\deg}\mathrm{Var}(t)+r_{\mathrm{prod}}(2-e^{-r_{\deg}t})+{\mathrm{GFP}}_{0}r_{\deg}e^{-r_{\deg}t}$$

$$\mathrm{after}\;t_{\mathrm{delay}}:\;\frac{\partial \mathrm{Var}(t-t_{\mathrm{delay}})}{\partial t}=-2r_{\deg}\mathrm{Var}(t-t_{\mathrm{delay}})+r_{\deg}\mathrm{GFP}(t_{\mathrm{delay}})e^{-r_{\deg}(t-t_{\mathrm{delay}})},$$

with solutions

$$\mathrm{before}\;t_{\mathrm{delay}}:\;\mathrm{Var}(t)=\frac{1}{r_{\deg}}e^{-2r_{\deg}t}\left(r_{\deg}({\mathrm{GFP}}_{0}(e^{r_{\deg}t}-1)+{\mathrm{Var}}_{0})+r_{\mathrm{prod}}e^{r_{\deg}t}(e^{r_{\deg}t}-1)\right)$$

$$\mathrm{after}\;t_{\mathrm{delay}}:\;\mathrm{Var}(t-t_{\mathrm{delay}})=e^{-2r_{\deg}(t-t_{\mathrm{delay}})}(\mathrm{GFP}(t_{\mathrm{delay}})(e^{r_{\deg}(t-t_{\mathrm{delay}})}-1)+\mathrm{Var}(t_{\mathrm{delay}})),$$

where

$$\mathrm{Var}(t)=\frac{1}{r_{\deg}}e^{-2r_{\deg}t_{\mathrm{delay}}}\left(r_{\deg}({\mathrm{GFP}}_{0}(e^{r_{\deg}t_{\mathrm{delay}}}-1)+{\mathrm{Var}}_{0})+r_{\mathrm{prod}}e^{r_{\deg}t_{\mathrm{delay}}}(e^{r_{\deg}t_{\mathrm{delay}}}-1)\right)$$

Var_0_ = Var(0), the initial stochastic variance at time point 0, and GFP(t), GFP(t_delay_) as derived in the main text.

Together, the total variance of the Gaussian distribution accounting for measurement and intrinsic noise for time point t is given by

$${\mathrm{Var}}_{\mathrm{total}}(t)=\sigma^{2}+\mathrm{Var}(t).$$

The log-likelihood for the more complex, time-dependent, Gaussian noise model is then given by

$$\mathrm{logL}(\theta_{i})=\frac{-1}{2}\underset{k}{\sum }\log(2\pi {\mathrm{Var}}_{\mathrm{total}i}(t))+\frac{{\left({\bar{y}}_{i}^{k}-y(t_{k},\theta_{i})\right)}^{2}}{{\mathrm{Var}}_{\mathrm{total}i}(t)}.$$

We obtained the optimal model parameters of both models and both noise models for the total GFP traces for each cell by performing maximum likelihood estimation.

### Optimization and parameter estimation

For each total GFP trace separately for r1 and r2, we computed the model parameters for the models accounting and not accounting for repression. The initial total GFP GFP_0_, the basal production rate r_basal_, and the degradation rate r_deg_ are the model parameters for the model not accounting for repression. Instead of a basal production rate, r_basal_, we have a production rate, r_prod_, for the model accounting for repression. Also, we discovered the time point of the repression response delay t_delay_ for the model accounting for repression. For both models, we also inferred the noise parameter σ determining the spread of the Gaussian noise model and potentially the initial stochastic variance Var_0_ when testing the sophisticated noise model. Overall, this results in 4 (5) inferrable parameters for the model not accounting for repression and 5 (6) inferrable parameters for the model accounting for repression, assuming a time-(in)dependent noise model. We assumed that all parameters are constant over time. For numerical reasons we optimized the parameters in log_10_ scale [35] and rescaled the data by 10^7^. As total Gal1-GFP fluorescence signal and total Gal1-GFP molecules are (linearly) mapped by an unknown constant, the number of total Gal1-GFP molecules is always scalable by that unknown constant that we exploit to increase convergence. The lower and upper bounds for all initial, rate, and noise parameters are –10 and 1 in log_10_ scale, respectively, assuring that the whole range of biologically plausible parameter values is covered. The lower and upper bounds for the repression response delay are given by 36 s and 2 h (corresponding to –2 and log_10_(2) in log_10_ scale). As we only considered 2 h of glucose repression, we did not allow the time delay to take on larger values. We performed multi-start local optimization of the negative log-likelihood using the parameter estimation toolbox PESTO [36]. For each model and total GFP trace, we performed local optimization runs from at least 20 different Latin-hypercube-sampled starts. If less than five starts converged, i.e. the objective function values of the starts differ less than 0.1 to the best start, we re-ran the optimization with 50, 100, and 200 starts until at least five starts converged for each GFP trace.

### Model selection

We used the Bayesian information criterion (BIC) [37] for comparing the model accounting and not accounting for repression per total GFP trace. The BIC is calculated by

BIC=log(n)k‐2logL,

where n is the number of data points, k is the number of estimated parameters and logL is the log-likelihood value for the maximum likelihood estimate of the model parameters. Here, the number of estimated parameters is either four for the model not accounting for repression or five for the model accounting for repression. The BIC rewards high likelihood values and penalizes the model complexity in the form of additional model parameters. We considered the model accounting for repression to fit a given total GFP trace considerably better than the model not accounting for repression if BIC_repression_ < BIC_no repression_–10 (Figs 3B–3D, S1D and S1E).

### Statistical analysis

#### Comparison of initial total GFP of total GFP traces

On the estimated initial total GFP, GFP_0_, of all total GFP traces significantly better fitted by a model accounting for repression and all total GFP traces better fitted by a model not accounting for repression, we did a two-sided median test. To avoid false-positive results, we used the Bonferroni correction, which adjusts the significance-level ɑ = 0.05 by the total number of investigated null hypotheses m, such that ɑ’ = ɑ/m. In this study, the total number of null hypotheses for the analysis is m = 12:

- Two hypothesis tests comparing initial total GFP between fits of the models accounting and not accounting for repression for repressions r1 and r2 (main analysis),
- Four hypothesis tests comparing estimated single-cell parameters between repressions r1 and r2 (main analysis),
- Two hypothesis tests comparing initial total GFP between fits of the models accounting and not accounting for repression for repressions r1 and r2 (replicate analysis),
- Four hypothesis tests comparing estimated single-cell parameters between repressions r1 and r2 (replicate analysis).

### Comparison of estimated single-cell parameters between repressions r1 and r2

We ignored all traces that were well described by a model not accounting for repression (uninduced cells) and focused the statistical analysis on the total GFP traces for which the model accounting for repression gave a considerably better fit (see the Model selection, Figs 3B–3C and S1D). For those total GFP traces, we compared the estimated single-cell parameters of initial total GFP, GFP_0_, repression response delay t_delay_, production and degradation rates, r_prod_ and r_deg_, for repressions r1 and r2. We performed a two-sided paired sign test on the estimated single-cell parameters of paired mother cells in repressions r1 and r2 (Figs 4 and S1G). To avoid false-positive outcomes, we used the Bonferroni correction, which modified the significance level of ɑ = 0.05 by the total number of tested null hypotheses m to ɑ’ = ɑ/m, with m = 12.

### Comparison of estimated single-cell parameters between the time-independent and time-dependent noise models

We compared the estimated single-cell parameters using a paired-sample t-test and corrected for false-positive outcomes by applying the Bonferroni correction with m = 8 (one null hypothesis for each estimated single-cell parameter = 4, for both repressions r1 and r2 = 8).

### Implementation and data/code availability

The data and MATLAB code corresponding to this manuscript are available under https://github.com/marrlab/Gal1repression.

## Supporting information

S1 Fig Shortened repression delay in repression r2 at the single-cell level for replicate experiment. (A) Single-cell traces of total Gal1-GFP fluorescence signal across two inductions i1 and i2 (gray) and repressions r0, r1, and r2 (blue). (B) Comparison of means of the normalized total Gal1-GFP fluorescence signals of repressions r1 and r2. (C) Single-cell traces of total GFP signal adjusted for dilution for the first two hours of repression r1 (left) and repression r2 (right). Time to maximal mean total GFP is 24 min shorter in repression r2, where mean total GFP is indicated by the dotted line and the maximal mean total GFP is highlighted by the dot. Bootstrap (10^5^) samples were drawn to generate mean ± std. (D) Ten exemplary total GFP traces (dotted lines) and best fits (solid lines) for repressions r1 (left) and r2 (right). GFP traces best fitted with a model accounting for repression are shown in black and fits of total GFP traces best fitted with a model not accounting for repression are shown in red. (E) The median initial total GFP, GFP_0_, is higher in traces better fitted by the model accounting for repression (black) than in traces better fitted by the model not accounting for repression (red). This confirms that the model accounting for repression fits induced cells better, while the model not accounting for repression fits uninduced cells. The number of cells and percentages of all GFP traces best fitted by the model accounting for repression and model not accounting for repression are shown. (F) Time to maximal mean total GFP is decreased in repression r2 for cells with repression kinetics (0.97 ± 0.08 vs. 0.88 ± 0.04). Bootstrap (10^5^) samples of the cells with repression kinetics were drawn to generate mean ± std. (G) Comparison of paired estimated single-cell parameters of cells with repression kinetics of repression r1 and r2 shows that the median initial total GFP, GFP_0_, and median repression delay, t_delay_, are significantly different (*p* = 4.6·10^−7^ and *p* = 3.3·10^−3^, respectively, two-sided paired sign test correcting for multiple testing with Bonferroni correction, m = 12, and the number of paired cells = 31), with median GFP_0_ increased and median t_delay_ decreased (median t_delay_ values of 0.83 and 0.53 for r1 and r2, respectively).
(TIF)

S2 Fig Estimated repression response delays are comparable between time-independent and time-dependent noise models for repressions r1 and r2. (A-B) The time-dependent noise model detects fewer cells with repression kinetics in repressions r1 (A) and r2 (B). However, the set of repressor cells detected across the two noise models is largely overlapping. (C-D) The estimated single-cell repression response delays are comparable (paired-sample t-test with Bonferroni correction, m = 8, and the number of paired cells = 40 for r1 and = 162 for r2) across both noise models for both repressions r1 (C) and r2 (D).
(TIF)

S3 Fig Estimated repression response delay is independent of GFP degradation rate. Total GFP traces of four simulated cells with varying degradation rates, r_deg_ = 0.5 h^-1^ (fast), r_deg_ = 0.2 h^-1^ (slow), r_deg_ = 0.04 h^-1^ (super slow), and r_deg_ = 0 h^-1^ (none) using Gillespie’s stochastic simulation algorithm and simulation parameters GFP_0_ = 100, t_delay_ = 0.5 h, r_prod_ = 100 h^-1^, and σ = 5. Performing parameter estimation and model selection, only the cell with no degradation (right) is misclassified as a cell without repression kinetics (with BIC_no repression_ < BIC_repression_). The repression response delay is correctly estimated to approximately 0.5 h for all simulated cells independent of the GFP degradation rate.
(TIF)
