# Supplementary figures and images for: Altered expression response upon repeated gene repression in single yeast cells
Source: PLoS Comput Biol. 2022 Oct 18;18(10):e1010640. doi: 10.1371/journal.pcbi.1010640 (PMC9633002; doi:10.1371/journal.pcbi.1010640)

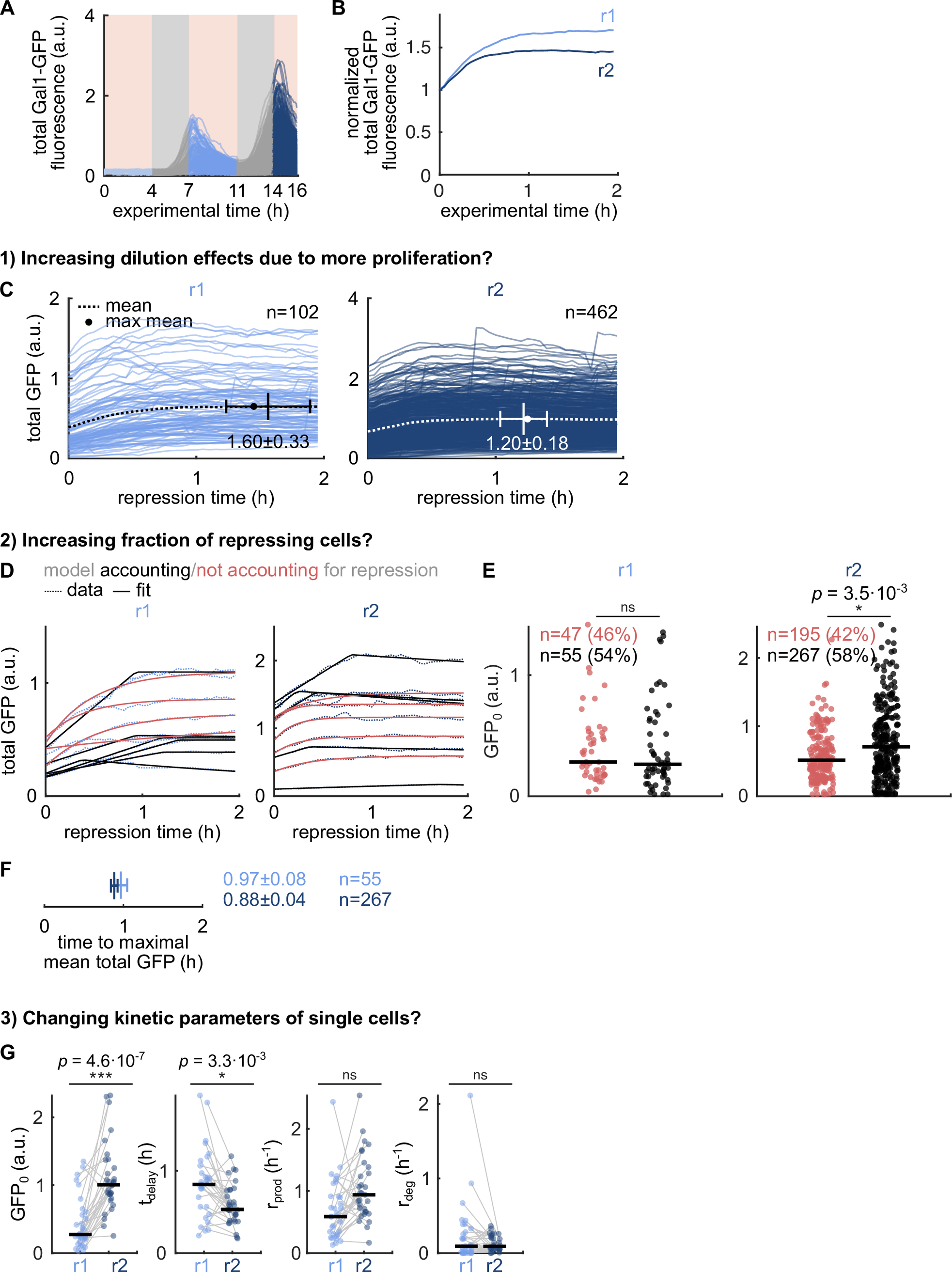

Supplement: S1 Fig — (A) Single-cell traces of total Gal1-GFP fluorescence signal across two inductions i1 and i2 (gray) and repressions r0, r1, and r2 (blue). (B) Comparison of means of the normalized total Gal1-GFP fluorescence signals of repressions r1 and r2. (C) Single-cell traces of total GFP signal adjusted for dilution for the first two hours of repression r1 (left) and repression r2 (right). Time to maximal mean total GFP is 24 min shorter in repression r2, where mean total GFP is indicated by the dotted line and the maximal mean total GFP is highlighted by the dot. Bootstrap (105) samples were drawn to generate mean ± std. (D) Ten exemplary total GFP traces (dotted lines) and best fits (solid lines) for repressions r1 (left) and r2 (right). GFP traces best fitted with a model accounting for repression are shown in black and fits of total GFP traces best fitted with a model not accounting for repression are shown in red. (E) The median initial total GFP, GFP0, is higher in traces better fitted by the model accounting for repression (black) than in traces better fitted by the model not accounting for repression (red). This confirms that the model accounting for repression fits induced cells better, while the model not accounting for repression fits uninduced cells. The number of cells and percentages of all GFP traces best fitted by the model accounting for repression and model not accounting for repression are shown. (F) Time to maximal mean total GFP is decreased in repression r2 for cells with repression kinetics (0.97 ± 0.08 vs. 0.88 ± 0.04). Bootstrap (105) samples of the cells with repression kinetics were drawn to generate mean ± std. (G) Comparison of paired estimated single-cell parameters of cells with repression kinetics of repression r1 and r2 shows that the median initial total GFP, GFP0, and median repression delay, tdelay, are significantly different (p = 4.6·10−7 and p = 3.3·10−3, respectively, two-sided paired sign test correcting for multiple testing with Bonfe [file pcbi.1010640.s001.tif]

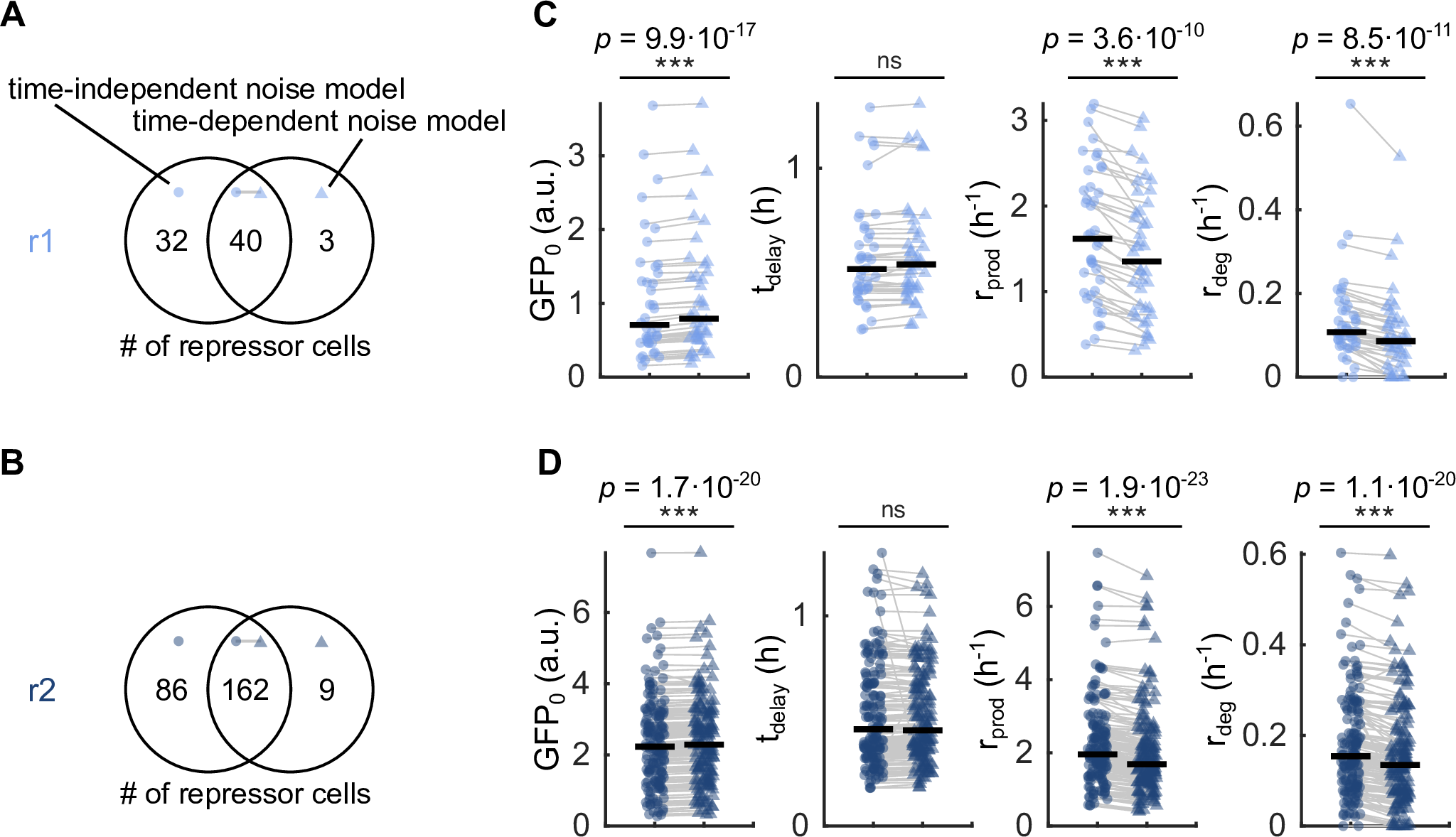

Supplement: S2 Fig — (A-B) The time-dependent noise model detects fewer cells with repression kinetics in repressions r1 (A) and r2 (B). However, the set of repressor cells detected across the two noise models is largely overlapping. (C-D) The estimated single-cell repression response delays are comparable (paired-sample t-test with Bonferroni correction, m = 8, and the number of paired cells = 40 for r1 and = 162 for r2) across both noise models for both repressions r1 (C) and r2 (D). (TIF) [file pcbi.1010640.s002.tif]

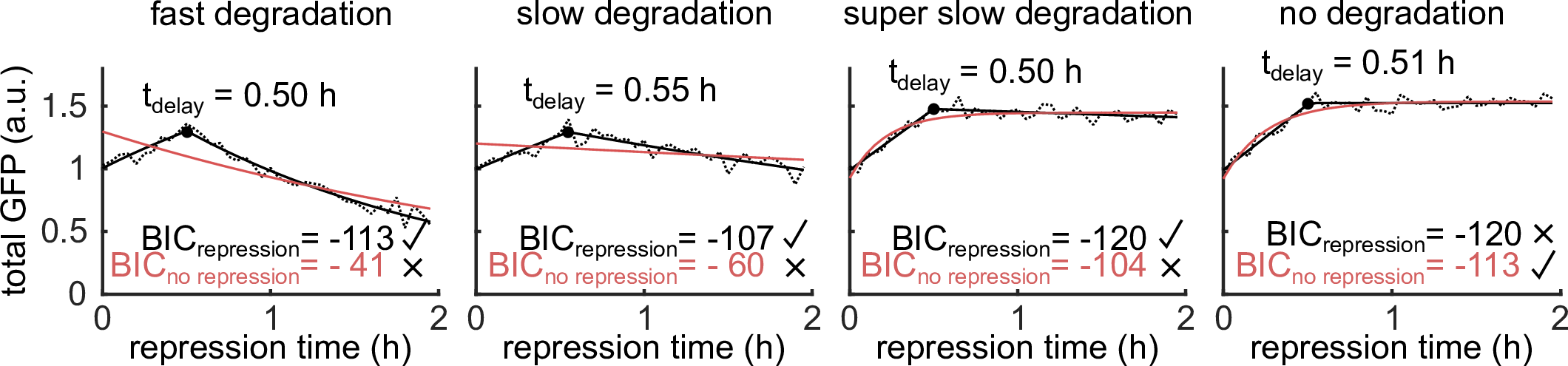

Supplement: S3 Fig — Total GFP traces of four simulated cells with varying degradation rates, rdeg = 0.5 h-1 (fast), rdeg = 0.2 h-1 (slow), rdeg = 0.04 h-1 (super slow), and rdeg = 0 h-1 (none) using Gillespie’s stochastic simulation algorithm and simulation parameters GFP0 = 100, tdelay = 0.5 h, rprod = 100 h-1, and σ = 5. Performing parameter estimation and model selection, only the cell with no degradation (right) is misclassified as a cell without repression kinetics (with BICno repression < BICrepression). The repression response delay is correctly estimated to approximately 0.5 h for all simulated cells independent of the GFP degradation rate. (TIF) [file pcbi.1010640.s003.tif]
